# Estimation of Atmospheric Path Delays in TerraSAR-X Data using Models vs. Measurements

**DOI:** 10.3390/s8128479

**Published:** 2008-12-19

**Authors:** Michael Jehle, Donat Perler, David Small, Adrian Schubert, Erich Meier

**Affiliations:** 1 Department of Geography, University of Zurich, Winterthurerstrasse 190, CH-8057 Zurich, Switzerland; 2 Institute of Geodesy and Photogrammetry, ETH Zurich, HPV G56, Schafmattstrasse 34, CH-8093 Zurich, Switzerland

**Keywords:** Synthetic aperture radar, SAR, atmosphere, troposphere, ionosphere, path delay, geolocation accuracy, calibration, TerraSAR-X

## Abstract

Spaceborne synthetic aperture radar (SAR) measurements of the Earth's surface depend on electromagnetic waves that are subject to atmospheric path delays, in turn affecting geolocation accuracy The atmosphere influences radar signal propagation by modifying its velocity and direction, effects which can be modeled. We use TerraSAR-X (TSX) data to investigate improvements in the knowledge of the scene geometry. To precisely estimate atmospheric path delays, we analyse the signal return of four corner reflectors with accurately surveyed positions (based on differential GPS), placed at different altitudes yet with nearly identical slant ranges to the sensor. The comparison of multiple measurements with path delay models under these geometric conditions also makes it possible to evaluate the corrections for the atmospheric path delay made by the TerraSAR processor and to propose possible improvements.

## 1. Introduction

The correction of atmospheric path delays in high-resolution spaceborne synthetic aperture radar systems has become increasingly important with continuing improvements to the resolution of SAR systems surveying the Earth. Atmospheric path delays must be taken into account in order to achieve geolocation accuracies better than 1 meter. These effects are mainly due to ionospheric and tropospheric influences. Path delays through the ionosphere are frequency-dependent, proportional to the inverse square of the carrier [1, 2]. At frequencies higher than L-band under average solar conditions, the major contribution of the atmospheric path delay comes from the troposphere [2, 3]. The tropospheric delay is usually divided into hydrostatic, wet and liquid components [4]. The hydrostatic delay is mainly related to the dependency of the refractive index on the air pressure (i.e. target altitude) and the wet delay on the water vapour pressure. The liquid delay is due to clouds and water droplets. While the wet component can be highly variable, the hydrostatic delay normally only changes marginally because of the lack of significant pressure variations within the extent of a typical SAR scene [4].

Interferometric radar meteorology produces high resolution maps of integrated water vapour for investigations in atmospheric dynamics and forecasting [4]. Using that knowledge, global and local atmospheric effects (e.g. vortex streets, heterogeneities, turbulences) can be detected or even removed using interferometric and multi-temporal data [5–7], or by inclusion of global water vapour maps from the ENVISAT Medium Resolution Imaging Spectrometer (MERIS) sensor [8]. In addition to interferometric applications, there is a growing interest in the correction of atmospheric influences within a single SAR image. Especially for SAR geolocation measurements, these atmospheric contributions introduce 'geolocation noise' that without correction causes shifts in geocoded products.

In this paper, the tropospheric path delay was assumed to depend only on the target's altitude and the local incidence angle of the radar wave. As the variability of the wet path delay is within ≈0.3 m [4], the wet delay in the model is based on average atmospheric conditions, maintaining the height-and incidence angle dependencies. Thus, the contribution of the wet component to the geolocation error should usually be significant below < 0.15 m. For comparison and as a reference model, a ray-tracing approach using current weather data is introduced. A set of TSX data and GPS measurements are used to verify the results from the model, as well as for comparison with the operational TSX processor's own atmospheric correction factors. The ionospheric contributions are estimated using TEC estimates from the GNSS network, and are compared to the DLR processor estimates provided in the TSX products. Since the TSX operational processor corrects the whole scene in question for the influence of the atmosphere using average TEC values, the mean scene height and the nominal mid-range incidence-angle [9], atmosphere-induced geolocation errors of ≈1 m are possible in mountainous regions. Together with DGPS measurements of four on-site corner reflectors and the TSX data, the results from the models and the measurements were cross-validated. A set of six TSX scenes were used to compare the operational 'average' atmospheric correction to a model utilizing meteorological data, as well as to a simple altitude-dependent model. While the meteorological model may not be suitable for operational use, the altitude-dependent model is straightforward and easy to implement. A comparison between these approaches and the DGPS measurements indicates a path toward improvement, especially in mountainous areas.

## 2. Methodology

**Figure 1.**
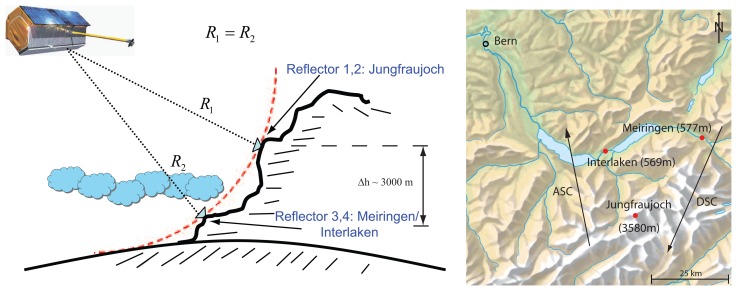
Observation geometry.

In the following, six TerraSAR-X Stripmap scenes (30 km × 20 km) containing four identical corner reflectors at altitudes of ≈570 m (Meiringen/Interlaken) and ≈3580 m (Jungfraujoch) were examined. Figure 1 illustrates the geometry and location of the scenes. In order to obtain nearly identical ranges for reflectors at different off-nadir angles, the reflectors closer to nadir are located ≈3000 m below the reflectors farther from nadir. Locations fulfilling these requirements were found in Switzerland for the descending case with a pair covering the Jungfraujoch and Meiringen regions, and for the ascending case with a Jungfraujoch and Interlaken pair. The arrangement serves two purposes:

(1) The same nominal antenna gain pattern correction is normally applied to two equal-range reflectors. Therefore, differences in their reflected intensities indicate topography-induced antenna gain pattern correction errors (not investigated within this paper).
(2) The nominal correction scheme for the atmospheric path delay can be tested by comparing predicted and measured ranges. The range differences between the high- and low-altitude reflectors help quantify *relative* differences in the path delay.

Another interesting side effect is that the average scene height in both configurations is close to the midpoint between the two reflector altitudes. Additional meteorological data (temperature, water vapour pressure, air pressure) from weather stations near Meiringen, Interlaken and Jungfraujoch provided further reference information for accurate modeling of the refractive index and atmospheric path delays.

Though they play only a minor role in this case, ionospheric path delays observed during the data takes and at the corresponding locations were estimated using the total electron content (TEC) along the ray path. TEC measurements were obtained from global vertical TEC maps with bi-hourly temporal resolution. The TEC maps can be downloaded in the IONosphere map EXchange format (IONEX) from the Center for Orbit Determination in Europe (CODE) [10].

## 3. Models and Measurements

The following sections provide a brief description of two models used: (a) Raytracer, and (b) height-dependent. In addition, the measurements made for the estimation of the atmospheric path delays are described. While the raytracer uses weather data for an estimation of the path delays with mm accuracy the altitude-dependent approach should provide a simplified model to correct path delays with cm accuracy.

### 3.1. Raytracer

The tropospheric delay is estimated on the basis of data provided by a numerical weather model [11]. Using this information, the raytracing algorithm integrates through the refractivity field along the path between the satellite and the point on the surface of the Earth.

The non-hydrostatic local area model COSMO-2 is used as a numerical weather model. It is operated by the Swiss Federal Office of Meteorology and Climatology and covers central Europe. It has a resolution of about 2 km and consists of 60 layers. The bottom layer follows the terrain, while the top boundary ends at 23589 m above the reference ellipsoid (WGS84). The model is used for the determination of the refractivity.

The raytracer assumes that the path followed by the ray is equivalent to the shortest geometrical path between the satellite and the point of interest. It is therefore only determined by the satellite position and the point of interest, but not by the refractivity field. This permits a simple computation of the ray paths. Since the refractivity field and its variability decreases with altitude, the length of the integration steps can be enlarged at upper levels without significantly reducing the accuracy, saving computation time. The integration method used is Newton-Cotes quadrature. However, this method has a fixed integration step length. To overcome this constraint, the atmosphere is subdivided into layers, each with a characteristic integration step size. By fixing the number of sampling points and increasing the thickness of a layer, the section of the ray path within the layer is lengthened. Consequently, the step size is increased, slightly reducing the accuracy. The thicknesses of the layers are chosen to cause zenith path delays nearly equivalent to those from a standard refractivity atmosphere (see Equation 2 and [12]). The boundaries of the layers are computed by following the recursive formula

(1) $$h_{i+1}=-h_{s}\log(\exp(-\frac{h_{i}}{h_{s}})-\frac{{\text{dt}}_{\text{tot}}}{n})$$

(2) $${\text{dt}}_{\text{tot}}=\int_{h_{0}}^{h_{n}}\exp(-\frac{h}{h_{s}})\text{dh}$$

where *h_i_* is the lower boundary height of the *i*-th layer (*i* = 0,…, *n* − 1) and *h_s_* the scale height. The parameter *h*_0_ is set to the height of the point of interest and *h_n_* to the height up to which will be integrated. The integration is carried out for each layer, and then the delays for all layers are added up to obtain the total result.

The refractivity N is usually not a prognostic variable in numerical weather models. However, it can be calculated from the partial dry air pressure *p_d_* (in hPa), temperature *T* (in K) and partial water vapour pressure *p_w_* (in hPa) using a formula published by Rüeger [13]

(3) $$\text{N}=77.6890\frac{p_{d}}{T}+71.2952\frac{p_{w}}{T}+375463\frac{p_{w}}{T^{2}}.$$

The prognostic variables used in the integration are interpolated at the sampling points. A multi-linear interpolation method is applied in 4 dimensions (space and time). If a sampling point is located outside the domain of the weather model, the meteorological quantities are extrapolated from the values at the boundaries. Points situated above or below the area of interest are exponentially extrapolated, whereas points located adjacent to the area of interest are set to the value of the nearest boundary point.

The values of the interpolation parameters are listed in Table 1. The free parameter *h_s_* in the exponential function used for estimating the layer thickness is proposed in [12]. The upper bound of the accuracy of the integration algorithm is set to 1 mm. Several tests were carried out to find adequate values for the remaining parameters fulfilling the accuracy constraints. More details on the accuracy of raytracers can be found in [14, 15].

### 3.2. Height-dependent Model

Since the tropospheric delay is most sensitive to altitude, a quick, straightforward and purely height-dependent approach was derived from standard models including mean estimates of the surface air-pressure *P_0_*, temperature and water vapour. As mentioned in the introduction, the tropospheric delay is usually divided into hydrostatic- (Ψ_hyd_), wet- (Ψ_wet_) and liquid- (Ψ_liq_) components and can be written as [16]:

(4) $$\Psi_{\text{tropo}}=\Psi_{\text{hyd}}+\Psi_{\text{wet}}+\Psi_{\text{liq}}$$

The hydrostatic component refers to a standard atmosphere (in hydrostatic equilibrium). The wet component accounts for the water vapour while the liquid component takes into account the liquid water content (clouds, droplets) along the signal path. Due to its small contribution (on the order of a mm) Ψ_liq_ is usually neglected for SAR path delay estimates [4]. The hydrostatic component Ψ_hyd_ in the nadir direction can be derived from [17, 18]:

(5) $$\Psi_{\text{hyd}}=10^{-6}k_{1}\cdot \frac{R_{d}}{g_{m}}P_{0}.$$

where *g_m_* is the acceleration due to local gravity, $k_{1}=77.6\;[\frac{K}{\text{mbar}}]$ is a refractive constant, and $R_{d}=287\;[\frac{J}{K.\text{kg}}]$is the ideal gas constant The wet delay contribution is estimated using [19]:

(6) $$\psi_{\text{wet}}=10^{-6}\cdot (\frac{(k_{2}^{\prime }T_{m}+k_{3})R_{d}e_{0}}{T_{0}(g_{m}(\lambda +1)-\beta R_{d})})\cdot \kappa_{\text{wet}}$$

with:

(7) $$\kappa_{\text{wet}}={\left(1-\frac{\beta h}{T_{0}}\right)}^{\frac{(\lambda +1)gm}{R_{d}\beta }-1}$$

where $k_{2}^{'}=23.3\;[\frac{K}{\text{mbar}}]$, $k_{3}=3.75\cdot 10^{5}\;[\frac{K^{2}}{\text{mbar}}]$ are refractive constants, $\beta =6.5\;[\frac{K}{\text{km}}]$ is the temperature lapse rate, *T*_0_ [K] the temperature-, *e*_0_ [hPa] the water vapour pressure above sea level, *T_m_* [K] the mean temperature of water vapour, *h* the target height and *λ* [unitless] the average water vapour decrease.

**Figure 2.**
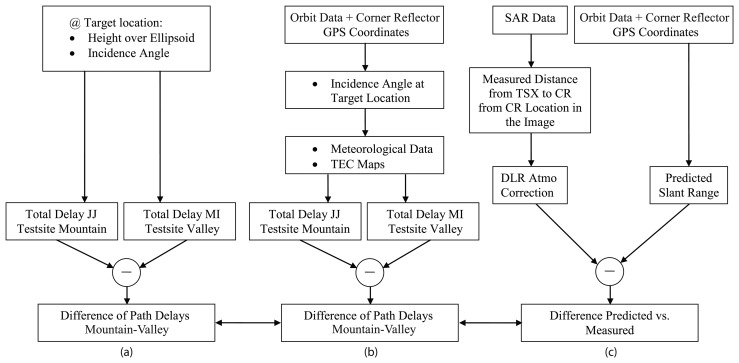
Scheme of methods used for comparison of atmospheric path delays; Testsite Jungfraujoch (JJ) on mountain site, Meiringen and Interlaken (MI) in the valley.

For the generation of a single altitude-dependent estimate of the tropospheric path delay for SAR applications, the average atmospheric parameters shown in Table 2 are used to determine the coefficients of a polynomial that fits the path delay data for heights h ranging from 0 to 9000 m in a least-squares sense. The resulting approach

(8) $$\Psi_{\text{tropo},\text{zenith}}=\frac{h^{2}}{8.55.10^{7}}-\frac{h}{3411}+2.41\;[\text{m}]$$

estimates the tropospheric delay in the zenith direction. The delay in the look direction of the antenna can be approximated using the nominal incidence angle *α_inc_* [rad] according to

(9) $$\Psi_{\text{tropo}}=\frac{\Psi_{\text{tropo},\text{zenith}}}{\cos\;\alpha_{\text{inc}}}\;[\text{m}],$$

where the incidence angle is calculated using the local height above the ellipsoid. This approximation of tropospheric path delay is compared later with an average delay correction and a path delay estimation based on the raytracer using meteorological data as described above. Figure 2 shows a block diagram of the three methods used for the estimation of tropospheric path delays. Figure 2a) illustrates the altitude-dependent method, Figure 2b) the raytracer and Figure 2c) the estimation and comparison of path delays from SAR data and GPS measurements described in the following section (JJ: Jungfraujoch, MI: Meiringen/Interlaken).

### 3.3. SAR and GPS Measurements

Estimation of atmospheric path delay directly from the SAR data was performed using precise DGPS measurements of four observed targets (corner reflectors) in the images. The range distance between the DGPS coordinates and the sensor is considered to be the reference for all estimates. The atmospheric corrections proposed in the TSX annotations, based on an average reference height (AVG), are applied to the image data (correction of fast time parameters). Their differences compared to the GPS measurements provide relative deviations from the reference range distance. The relative differences in path delay estimates between the corner reflectors located at different altitudes are calculated in a final step. These calculations point out varying propagation properties and indicate possibilities for refinement (Figure 2c). Ionospheric contributions are also considered, although they play a minor role at X-band frequencies, especially at the current solar minimum. Average estimates for their contribution (path delay at 5 TECU) are provided in the TSX product annotations [20]. Measured values were estimated using total electron content (TEC) maps describing spatial variations in TEC values across the Earth. The one-way ionospheric path delay may be estimated from [21]:

(10) $$\psi_{\text{iono}}=K.\frac{\text{TEC}}{f_{c}^{2}}\cdot \frac{1}{\cos\alpha_{\text{inc}}}$$

where *f_c_* is the center frequency of the radar wave, *c* the speed of light and $K=40.3\frac{{\text{m}}^{3}}{{\text{s}}^{2}}$ is a refractive constant. The factor $\frac{1}{\cos\alpha_{\text{inc}}}$ converts the path delay from nadir to the path at a particular incidence-angle. Measurements from GNSS networks provide multiple maps per day, and may be downloaded in a standardized format from the internet [10]. Given the TEC values together with the corresponding satellite and target positions, the expected path delay is calculated.

## 4. Path Delay Results

**Figure 3.**
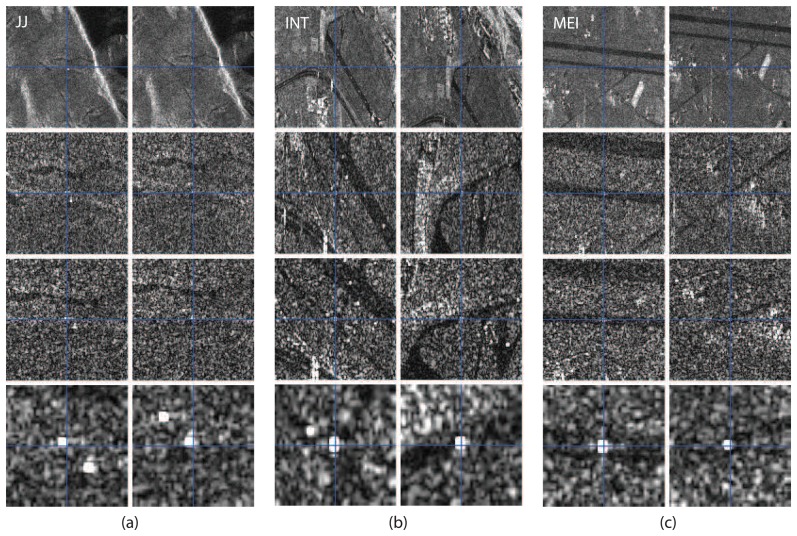
Example of zoomed in and interpolated corner reflectors in TSX imagery. The blue cross indicates the GPS derived position prediction and the strong white target the actual measured position of the CR in the image.

**Figure 4.**
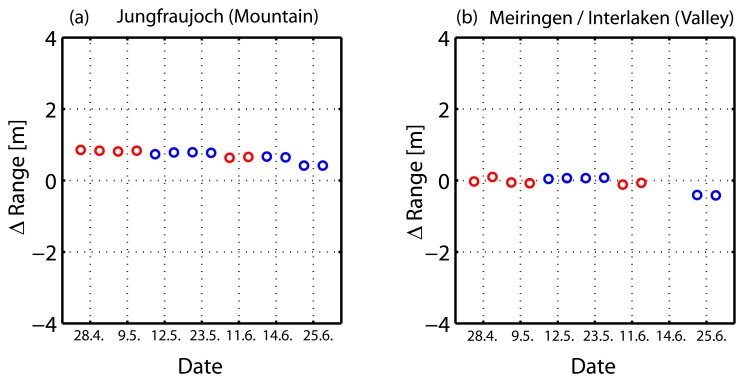
Absolute location error estimations for each testsite. Red circles mark descending and blue circles ascending geometry. Note: An additional data take is plotted in Figure 4(a). As it was not possible to deploy the CRs in Interlaken at that time, this data take was omitted in the analysis that followed.

As a first test, the absolute image localization error for all four corner reflectors in each of the six TSX products was measured. Accurately surveyed DGPS measurements of the corner reflectors were used to predict their range and azimuth positions in each image, and these predictions were compared to their measured locations. Figure 3 shows an example for the absolute location error estimate for (a) Jungfraujoch (JJ), (b) Interlaken (INT) and (c) Meiringen (MEI). At successive 'zoom' levels, the blue crosses indicate the prediction based on the GPS measurements, which represent the runtime measurements under ideal vacuum conditions. These image position predictions were made by solving the Doppler and range equations [22] using the surveyed target coordinates, together with the precise orbit state vectors and the image timing annotations [20]. The precise corner reflector (phase center) position in the image was determined by searching for local maxima in the neighborhood of the strong targets, using complex-FFT oversampling (factor of 50) to obtain sub-sample accuracy [22]. Results of the following analysis are based on the location error of the two corner reflectors at each test site, both at the same altitude and equidistant to the sensor. Figure 4 shows a scatter plot of all estimated location errors. The blue circles indicate descending-, the red circles ascending products. While for Interlaken and Meiringen (b) the range errors are on the order of a cm, for the Jungfraujoch site (a) the range errors increase to a mean of approximately 0.58 m. Since the TSX tropospheric correction is based on an *average* scene height roughly halfway between the test site altitudes (Jungfraujoch and Meiringen/Interlaken), the expected average location errors in range for the sites would be expected be approximately of the same magnitude with opposite signs.

**Figure 5.**
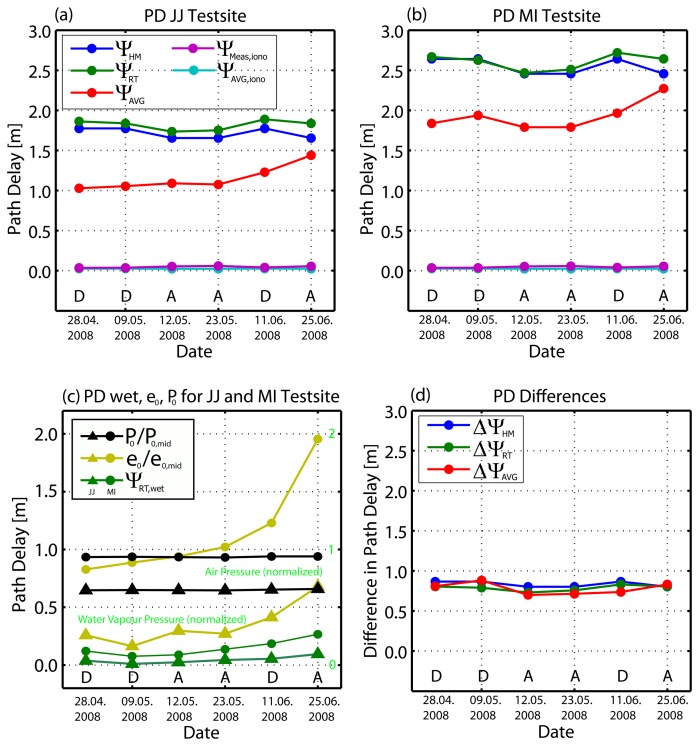
Modelled and measured atmospheric path delays. Path delays in a) for JJ testsite and in b) for the MI testsites. In c) wet path delays compared to measured air- and water vapour pressure (normalized) and in d) path delay differences between the results of the JJ and the MI testsites.

In order to estimate the height-dependent path delays for each test site, the range errors were subtracted from the average range delay (see Table 3). As expected, atmospheric delays at the higher altitude of the Jungfraujoch site (Ψ_JJ,AVG_) are smaller than at the Meiringen/Interlaken sites (Ψ_MI,AVG_). The estimated relative difference in one-way path delay between both sites has a mean of 0.779 m. Differences in the descending case are usually higher in comparison to the ascending cases, as the signal path through the troposphere was longer, due to the more oblique incidence angle. Results from the height-dependent model (HM) and the raytracer (RT) use the altitudes and incidence angles estimated for each corner reflector position. The path delays from the TSX annotations (∅Ψ_AVG_) are used together with the differences ΔRg._JJ_ and ΔRg._MI_ to map to the delays according to the altitude of the testsite. Figure 5(a) and (b) show the total tropospheric path delays (hydro + wet component) estimated from the models, including the results from the measurements as well as the ionospheric delays. In Figure 5(c) the black and yellow lines show the distribution of air pressure and water vapour during the data takes in comparison to the wet path delays estimated from the raytracer. Both pressure parameters are normalized to the assumptions of the standard atmosphere made in Table 2. Figure 5(d) shows the differences in atmospheric path between the mountain (Jungfraujoch) and the valley (Meiringen/Interlaken) testsites and therefore measures the dynamic of the path delay models at these altitudes. The results from the height-dependent model are very similar to the raytracer results. An exceptional scene was the data from June 25th (heavy rain) where high water vapour pressure was measured, which significantly increased the wet path delay contribution. The path delays estimated from the image as compared to the GPS measurements are plotted in red, and are less consistent with the model results, but strongly correlate with the water vapour measurements (Figures 5(a) to (c)). This is unsurprising, as the variations arise from the differences between these constant delays and the GPS-measured vacuum propagation (ΔRg._MI_, ΔRg._JJ_). The TSX average correction does not significantly vary across the ascending/descending geometries and therefore causes only a constant shift. From Figure 5(a) and (b) it can be seen that the shifts between the model and the measurements are nearly constant. This suggests that the tropospheric path delays from the TSX annotations consistently *underestimate* the true delays. On the other hand, this shift implies that if the delays from the models were applied to the TSX image, we would expect an average range shift ΔRg._MI_ and ΔRg._JJ_ of ≈0.7 m across all test sites. It follows that the measurements in Interlaken only performed with cm accuracy since the underestimated path delay having been compensated by the overall constant ≈0.7 m shift.

Path delays caused by the ionosphere were modelled to have been in the cm range. The average one-way ionospheric delays from measurements are comparable to, although slightly higher than, the average values provided in the TSX annotations (∅Ψ_Meas,iono_=4.7 cm, ∅Ψ_AVG,iono_=2.5 cm).

The relative differences of the path delays between the test sites in Figure 5(d) can be considered an indication of the model's ability to capture altitude-dependent variations. With mean values of ∅ΔΨ_HM_=0.822 m, ∅ΔΨ_RT_=0.786 m and ∅ΔΨ_AVG_=0.779 m, all models agreed closely.

## 5. Discussion and Conclusion

With TerraSAR-X, a civilian spaceborne satellite is for the first time able to observe the Earth with a radar resolution on the order of 1 m. At such resolutions, the influence of the atmosphere on the geolocation accuracy plays a significant role and must be taken into account. Various methods for the correction of these delays can be applied, depending on the scientific application. In this work, we investigated the range delays predicted by an altitude- and incidence-angle dependent model, a raytracer and a model which corrects the path delay as a scene average. Model results were compared for six TerraSAR-X datasets containing four corner reflectors, two at altitudes 3000 m above the others.

We found that the scene-average method significantly *underestimates* the tropospheric delays compared to the raytracer model and the height-dependent polynomial. While the range location errors for the low-altitude reflectors were normally on the order of a cm, the range errors at the high altitude station were usually over half a meter. The absolute location error for a scene-average path delay estimate should result in comparable range errors at equal height offsets above and below the average scene height. Our test sites at Jungfraujoch (3580 m) and Meiringen/Interlaken (≈570 m) with average scene heights of 2160 m/1860 m nearly fullfilled that condition. When the annotated delays from the TSX products were replaced by the results from the raytracer for the individual locations, a nearly constant range shift of ≈0.7 m was estimated. On the one hand, this indicates that the corrections from the raytracer are reasonable, yet on the other hand, suggests an inherent systematic shift of ≈0.7 m which is still within the specifications of the TSX accuracy requirements.

Since the dynamics of all methods are similar, differences between the models are likely due to different atmospheric starting positions. The results for the last two acquisitions are more similar for the various models. The range deviations from the image measurements and the path delays from the raytracer show that this is probably due to the increased amount of atmospheric water vapour during these data takes. As a result, the path delay in the SAR image increases. The standard correction from the TSX annotations does not take these effects into account. Therefore, the influence of the higher water vapour pressure could be directly observed in the changes in the range location errors. In other words, the larger path delay in the image compensates for the underestimated average path delay which leads to the observed 'increased' accuracy. Path delay estimates from the raytracer include the higher water vapour pressure, but probably to a lesser extent, as the heavy rainfall was observed to be very localized. This might also be seen from its path delay estimates. Loss of accuracy caused by poor modelling of wet path delay estimation in such storm events are not expected to exceed ≈10 to 15 cm.

Separation of the ionospheric delays from the total atmospheric delays also indicates that the average value from the TSX annotations tends to underestimate the delay estimated from the measured TEC maps by a mean of 2.2 cm. Although solar activity and therefore the ionospheric delay is presently near its minimum, future path delays at X-band in mid-latitude regions are not expected to regularly exceed 0.30 m.

Unlike the scene-average model, the results from the height-dependent polynomial are very close to the raytracer estimates. The height-dependent polynomial would therefore seem to be a straightforward alternative for operational use. Worse results would be expected at high latitudes, as the correction is based on a standard atmosphere most representative of mid-latitude regions. This could be improved relatively simply with extra terms 'capturing' latitude-induced dynamics. As the raytracer produces the most accurate results, a correction of the data with this approach would be desirable; however, operational inclusion of the current weather data is currently not feasible.

## Figures and Tables

**Table 1.** Setup parameters for the integration algorithm.

Parameter	Value
Scale height *h_s_*	7353.0 m
Integration height *h_n_*	60000 m
Number of layers *n*	12
Number of sampling points per layer	12

**Table 2.** Parameters used to model the tropospheric path delay.

**P**_0_*_,_*_mid_	**T**_0_*_,_*_mid_	**e**_0_*_,_*_mid_	*β*_mid_	*λ*_mid_
1013.25 [hPa]	288.15 [K]	11.691 [hPa]	6.5-10^−3^[K/m]	3 [-]

**Table 3.** Predicted path delays from measurements and GPS. The ∅Ref.Height, *α*_inc_ and ∅Ψ_AVG_ refer to the average scene height, the mid incidence angle and average tropospheric path delay respectively, as annotated in the TSX products (JJ: Jungfraujoch, MI: Meirin-gen/Interlaken, dates are all in the year 2008).

**Predicted Path Delay from Measurement and GPS**									

Date	A/D	∅Ref.Height	*α*_inc_	∅Ψ_AVG_	ΔRg._JJ_	ΔRg._MI_	Ψ_JJ,AVG_	Ψ_MI,AVG_	Ψ_JJ_-Ψ_MI_

28.04.	D	2163 m	31.2°	1.874 m	0.846 m	0.036 m	1.03 m	1.84 m	0.809 m
09.05.	D	2166 m	31.2°	1.873 m	0.818 m	-0.064 m	1.05 m	1.94 m	0.882 m
12.05.	A	1865 m	24.0°	1.845 m	0.755 m	0.055 m	1.09 m	1.79 m	0.700 m
23.05.	A	1827 m	24.0°	1.857 m	0.782 m	0.068 m	1.07 m	1.79 m	0.714 m
11.06.	D	2164 m	31.2°	1.874 m	0.646 m	-0.091 m	1.23 m	1.96 m	0.737 m
25.06.	A	1827 m	24.0°	1.857 m	0.418 m	-0.414 m	1.44 m	2.27 m	0.832 m

**Mean Values**				1.863 m	0.581 m	-0.068 m	1.16 m	1.92 m	0.779 m
